# Coping expectancies and disability across the new ICD‐11 chronic pain categories: A large‐scale registry study

**DOI:** 10.1002/ejp.1979

**Published:** 2022-05-31

**Authors:** Alice Munk, Henrik Børsting Jacobsen, Silje Endresen Reme

**Affiliations:** ^1^ The Mind‐Body Lab, Department of Psychology, Faculty of Social Sciences University of Oslo Oslo Norway; ^2^ Department of Pain Management and Research Oslo University Hospital Oslo Norway

## Abstract

**Background:**

Recently, a new classification system for chronic pain was included in the 11th edition of the International Classification of Diseases (ICD‐11). This study aims to investigate how expectancies of coping, that is pain catastrophizing and general self‐efficacy, are associated with ICD‐11 chronic pain categories in a large pain clinic population. Furthermore, we investigate how coping expectancies are associated with pain‐related disability, cross‐sectionally and longitudinally across the novel pain classifications.

**Methods:**

The sample was retrieved from the Oslo University Hospital Pain Registry and included baseline data from 2875 chronic pain patients and 12‐month follow‐up data for 920 patients. Demographic and clinical variables were compared across the ICD‐11 chronic pain categories through ANOVA. Multiple regression models were carried out to investigate cross‐sectional and longitudinal associations.

**Results:**

With the exception of age, our data showed no significant differences across the ICD‐11 chronic pain categories. Coping expectancies were associated with disability at baseline. At 12‐month follow‐up, coping expectancies did not predict pain‐related disability when controlling for baseline levels of disability, pain intensity and pain duration. Pain classification (primary vs secondary) did not contribute significantly to the models. Helplessness had the strongest simple relationship to disability, compared with global pain catastrophizing and its additional subscales, both cross‐sectionally and longitudinally.

**Conclusion:**

Coping expectancies, pain intensity and pain‐related disability appear similar across the novel chronic pain classifications, indicating that all pain patients may benefit from targeting these variables. Consistent with recent developments in stress theory, helplessness and self‐efficacy were cross‐sectionally associated with negative pain outcomes.

**Significance:**

Levels of coping expectancies, demographic characteristics, pain‐related disability and pain intensity are similar across all ICD‐11 chronic pain diagnostic categories. Thus, chronic primary pain is not stronger associated with psychosocial factors such as catastrophizing and self‐efficacy than chronic secondary pain. Therefore, chronic pain patients, independent of diagnosis, may benefit from the assessment of these psychosocial factors and targeted interventions such as CBT should be considered.

## 1 INTRODUCTION

Global estimates suggest that the prevalence of chronic pain is around 20% (Goldberg & McGee, 2011), with indications of increasing numbers (GDB 2016, 2017). Prevalence estimates depend on how chronic pain is classified, which affects health policy, research agendas, clinical practices and ultimately the patients. Recently, a taskforce under the International Association for the Study of Pain (IASP) developed a novel classification system for chronic pain, released as an integrated part of the 11th edition of the World Health Organization's International Classification of Diseases (ICD‐11) (World Health Organization, 2021). This new classification system goes into effect in 2022, and distinguishes between chronic primary and secondary pain syndromes. Here, chronic primary pain considers the pain a disease in itself, while chronic secondary pain attributes the pain to other underlying diseases.

Pain catastrophizing and self‐efficacy are well‐established risk‐ and resilience factors in chronic pain (Jackson et al., 2014; Martinez‐Calderon et al., 2019; Sullivan et al., 2001), but also represent two opposing expectancies of coping. While pain catastrophizing is characterized by helplessness, rumination and magnification (Sullivan et al., 2001), generalized self‐efficacy reflects a positive ability to cope with a wide range of challenging situations (Schwarzer & Jerusalem, 1995). In response to pain, people with a tendency to catastrophize might expect the pain to be overwhelming and uncontrollable, while people with high self‐efficacy expect their attempts to handle the pain to be successful. According to recent conceptualizations of stress theory (Munk et al., 2021; Ursin & Eriksen, 2004), these expectancies are labelled response outcome expectancies, and their role in regulating pain and stress is well established in both experimental and clinical data. Previous research have indeed shown that higher pain catastrophizing is positively correlated with pain intensity and pain‐related disability (Martinez‐Calderon et al., 2019; Sullivan et al., 2001), while general self‐efficacy is associated with a range of pain‐related outcomes in various clinical populations (Elden et al., 2016; Grasaas et al., 2020; Kawaguchi et al., 2020; Rashid et al., 2018; Schulz et al., 2015; Schwarzer et al., 2005).

The impact of pain catastrophizing and self‐efficacy on pain outcomes show similar effect sizes across different chronic pain syndromes (Jackson et al., 2014; Martinez‐Calderon et al., 2019), indicating that these cognitive factors are not condition‐specific. According to the new ICD‐11 classification, chronic primary pain is diagnosed when persistent pain is associated with significant emotional distress and/or severe functional disability (Nicholas et al., 2019). As emotional distress is a diagnostic criterion of chronic primary pain and not chronic secondary pain, it is interesting to investigate if psychosocial factors such as coping expectancies relate differently to the new ICD‐11 categories. Indeed, a previous study indicated lower levels of self‐efficacy and higher levels of helplessness and disability in patients with fibromyalgia (chronic primary pain) compared with arthritis (chronic secondary pain) (Moyano et al., 2019), while others do not find any such classification‐specific differences (Jackson et al., 2014).

Thus, we here aim to investigate how opposing expectancies of coping, hereunder pain catastrophizing and general self‐efficacy, relate to the new ICD‐11 classifications in a large pain clinic population. Additionally, we aim to investigate how expectancies of coping are associated with pain‐related disability, cross‐sectionally and longitudinally. Based on previous findings (Moyano et al., 2019; Treede et al., 2015), it is hypothesized that there are higher levels of catastrophizing and lower levels of self‐efficacy in chronic primary pain when compared to chronic secondary pain. We also hypothesized that lower self‐efficacy and higher pain catastrophizing are associated with higher levels of pain‐related disability cross‐sectionally and longitudinally, independent of ICD‐11 classification.

## 2 METHODS

### 2.1 Participants

The study is based on observational cross‐sectional and longitudinal registry data. All data were retrieved from the Oslo University Hospital Pain Registry (OPR) (Granan et al., 2019). The OPR is a comprehensive local quality registry from the largest outpatient pain clinic in Norway, covering over 60% of the Norwegian population. The clinic offers interdisciplinary assessment and treatment of patients with a variety of chronic pain conditions. As standard procedure, all patients complete a collection of self‐reported questionnaires prior to their first consultation. In the first consultation, the patient is thoroughly examined and diagnosed by a physician who specialized in pain medicine. Consenting patients receive an electronic follow‐up survey 12 months after their first consultation. During these 12 months, the majority of patients undergo individually targeted multimodal pain treatments. However, some patients receive only assessment and are referred back to their general practitioner or other relevant treatment alternatives. The interdisciplinary group of clinicians includes physicians, psychologists, nurses and physiotherapists. The type of treatments offered at the clinic cover a wide range of interventions, for example pharmacological treatment, invasive pain treatments, physical therapy and cognitive‐behavioural therapy.

The study population was determined by the number of patients who provided baseline data to the OPR in the period January 2017 to May 2020. Patients who provided baseline data to the registry outside this period had not had the opportunity to answer the follow‐up questionnaires either because the routine was not yet properly implemented in the first year of the OPR or because they had not reached the stage of their 12‐month follow‐up by the time the analyses for this study were performed. Therefore, they were not included in the study. The sample consists of patients diagnosed with ICD‐11 chronic primary or chronic secondary pain. In addition, patients diagnosed with ‘unspecified chronic pain’ (*n* = 307) were included in the sample while patients with the residual diagnostic category ‘other specified chronic pain’ (*n* = 17) were excluded. Neither ‘unspecified chronic pain’ nor ‘other specified chronic pain’ was directly relevant for the research question that focuses on differences between chronic primary and secondary pain conditions. Still, it was judged that the considerable group size of ‘chronic unspecified pain’ made it relevant to represent these patients in the study.

Baseline data are available from more than 90% of patients referred to the clinic. From January 2017 until May 2020, 2875 patients provided baseline data. Of these, approximately 77% consented to the follow‐up assessment, of which 42% responded (*n* = 920).

### 2.2 Measurements

An overview of the variables in the registry can be found here (Granan et al., 2019). The basic demographic data obtained were age, gender, marital status, education and employment status.

Pain classification was categorized according to the recent taxonomies ICD‐11 (World Health Organization, 2021). ICD‐11 divides chronic pain conditions into primary and secondary pain. Chronic primary pain is characterized as a disease in its own right and is associated with emotional distress and/or functional disability (Nicholas et al., 2019). Secondary chronic pain is further divided into six categories: secondary musculoskeletal pain, neuropathic pain, post‐surgical/post‐traumatic pain, cancer‐related pain, visceral pain, headache or orofacial pain (Treede et al., 2019). Finally, the residual diagnostic category ‘unspecified chronic pain’ was included in the study.

In the earliest version of the OPR, clinicians did not electronically register ICD‐11 codes. As such, ICD‐10 diagnoses were converted to ICD‐11 codes by experienced physicians and were based on a priori defined criteria. The physician in charge of the conversion process is a specialist in physical medicine and rehabilitation, with 19 years of clinical and research experience within the pain field and/or clinical registries. He is the head of the pain registry (OPR) and has been responsible for the clinical pain classification in the department of pain management and research in accordance with ICD‐10 since 2014, and both ICD‐10 and 11 since 2016. The conversion manual is available here (see Material S1).

### 2.3 Independent variables

Pain catastrophizing was measured with the Norwegian translation (Fernandes et al., 2012) of the original Pain Catastrophizing Scale (PCS) (Sullivan et al., 1995) which assesses catastrophic thoughts and emotions in response to pain. In the PCS, participants rate their responses on a 5‐point Likert scale ranging from ‘0; not at all’ to ‘4; all the time’. The PCS can be scored as one independent measure of pain catastrophizing, or as three independent subdomains: helplessness, magnification and rumination. The helplessness subscale includes items 1–5 and 12. The rumination subscale includes items 8–11. The magnification subscale includes items 6, 7 and 13. Examples of items of the three subscales are, for helplessness: ‘there is nothing I can do to control the pain’, for rumination: ‘I keep thinking about how much it hurts’ and magnification: ‘I wonder whether something serious might happen’. In the validation study of the Norwegian version of the PCS in chronic low back pain patients, a slightly different factor structure of the three subdomains occurred as a result of their factor analyses (Fernandes et al., 2012). However, as the authors conclude, these minor differences do not warrant a Norwegian version of PCS with a different factor structure than the original scale since it would make it impossible to compare subscores across studies. The internal consistencies of the scale were as follows: *α* = 0.90 for the full scale, *α* = 0.86 for the helplessness subdomain, *α* = 0.83 for the rumination subdomain and only *α* = 0.53 for the magnification subdomain (Fernandes et al., 2012). Since the internal consistency of the magnification subscale was not satisfactory, it cannot be recommended to use this as an independent instrument.

Self‐efficacy was assessed with the Norwegian version (Roysamb et al., 1998) of the General Self‐Efficacy Scale (GSE) (Schwarzer & Jerusalem, 1995). The GSE measures general self‐efficacy beliefs with the aim to predict coping with challenging demands and adaptation to stressful life situations. The scale consists of 10 items, for example ‘I can manage to solve difficult problems if I try hard enough’ and ‘I am confident that I could deal efficiently with unexpected events’ which are all rated on a 4‐point scale. The GSE has undergone satisfactory multicultural validation across a variety of healthy and clinical population groups including patients with cancer, heart disease and gastrointestinal diseases (Luszczynska, Gutiérrez‐Doña, et al., 2005; Luszczynska, Scholz, et al., 2005). The version used in this study has been validated in the Norwegian general population where the scale showed internal consistency at *α* = 0.92 (Bonsaksen et al., 2019).

### 2.4 Dependent variable

A measure of pain‐related function was obtained using a Norwegian translation and a slightly modified version of the Oswestry Disability Index (ODI) (Fairbank et al., 1980). The original ODI contains 10 items related to back pain and function. The modified ODI used in the present study is identical to the original with the exception that the word ‘back’ is removed from the introduction (Granan et al., 2019). The Norwegian version of ODI has been validated in pain patients showing internal consistency at *α* = 0.94 (Grotle et al., 2003).

A recent Cochrane review (Williams et al., 2020) concludes that psychological treatment can reduce pain‐related disability. As this study focuses on psychological factors related to chronic pain, this is the main rationale for choosing pain‐related disability as an outcome and not, for example pain intensity, which has been found to be more resistant to the effects of psychological treatment (Williams et al., 2020).

### 2.5 Confounding variables

Pain intensity was measured using a single question rated on a numeric rating scale (NRS): ‘How intense has your pain usually been’? The NRS is a continuous self‐report measure where respondents are asked to rate a single item on a 11‐point Likert scale (0–10). In pain research, NRS has proven strong validity and reliability in adult pain populations (Safikhani et al., 2018) and is less influenced by mood state (Jensen, 2003).

To attain a measure of pain duration, all participants were asked to report how long their pain has lasted in years and months.

### 2.6 Ethics and data protection

The regional committees for medical and health research ethics (REK) approved the present study (ref. 2021/254165).

Data from the OPR are not used for anything other than approved and regulated research projects, as described in the written informed consent. Patients who wish to withdraw their consent can do this at any time and without any consequences for follow‐up or treatment by contacting the responsible for the OPR. All data are encrypted and stored on a secure server that is only accessible to the leader of the OPR.

### 2.7 Statistical analyses

Descriptive statistical methods were used in order to characterize the study sample.

To assess our primary hypothesis on differences in psychosocial factors between the ICD‐11 diagnostic categories, we performed ANOVA's and Tukey's post hoc tests. All ANOVA's were performed with ICD‐11 diagnostic category as an independent variable (the variable includes a total of 8 groups).

To assess our second hypothesis, simple linear regression analyses were carried out to establish both cross‐sectional and longitudinal relationships between coping expectancies and disability at baseline and 12‐month follow‐up. Multiple regression models including partial eta squared effect sizes were used to confirm shared and unique contributions of coping expectancies to pain‐related disability.

Finally, differences in demographic variables, disability and coping expectancies between responders and non‐responders at the 12‐month follow‐up registration were investigated using independent t‐ and chi‐square tests.

Analyses were carried out in the IBM® SPSS® Statistics version 27.

## 3 RESULTS

### 3.1 Descriptive analyses

The average age of the participants was 49.4 years (range 17–95) and 57% were female. The patients had experienced pain for an average period of 7.5 years. The distribution of ICD‐11 chronic pain categories and a complete summary of the descriptive statistics are provided in Table 1.

**TABLE 1:** Summary of demographic variables, risk‐ and resilience factors and disability across ICD‐11 chronic pain categories

	All categories (*n* = 2799)	Chronic primary pain (*n* = 1469)	Chronic cancer‐related pain (*n* = 8)	Chronic postsurgical or posttraumatic pain (*n* = 178)	Chronic secondary musculoskeletal pain (*n* = 335)	Chronic secondary visceral pain (*n* = 38)	Chronic neuropathic pain (*n* = 456)	Chronic secondary headache or orofacial pain (*n* = 8)	Unspecified chronic pain (*n* = 307)
Categorical variables	%	%	%	%	%	%	%	%	%
Total	100	52.5	0.3	6.4	12	1.4	16.3	0.3	11
Female	58.2	58.5	62.5	59.8	57.3	63.2	58	75	55.5
Male	41.8	41.5	37.5	40.2	42.7	36.8	42	25	44.5
Living alone	30.6	30.4	25	28	32.5	44.4	29.4	28.6	31.8
Living with others	69.4	69.6	75	72	67.5	55.6	70.6	71.4	68.2
Working/student/military service	35.6	35	12.5	36.7	34.8	42.1	39	14.3	33.6
Not working	64.4	65	87.5	63.3	65.2	57.9	61	85.7	66.4
Comprehensive school (1–10 years)	15	15.5	12.5	11.5	16.6	18.9	13.4	0	15.1
Secondary school/vocational (11–13 years)	44.7	43.6	50	40.2	39.6	43.2	48.3	85.7	51.7
College degree (14–17 years)	31.4	32	37	35.6	34.7	32.4	29.2	14.3	25.7
Higher university (>17 years)	9	8.9	0	12.6	9.2	5.4	9.1	0	7.5

Continuous variables	Mean (SD)	Mean (SD)	Mean (SD)	Mean (SD)	Mean (SD)	Mean (SD)	Mean (SD)	Mean (SD)	Mean (SD)
Age (years)^1^	49.56 (15.77)	47.14 (15.26)	53.83 (18.61)	47.59 (15.04)	55.98 (16.68)	48.34 (16.37)	53.41 (15.52)	44.67 (23.58)	49.64 (14.57)
Pain duration (years)^2^	7.52 (7.41)	7.69 (7.42)	7.43 (6.71)	6.22 (6.03)	7.64 (7.61)	8.09 (7.74)	7.41 (7.53)	6.81 (5.73)	7.45 (7.75)
Pain intensity (NRS) (0–10)^3^	7.20 (1.79)	7.20 (1.80)	8.33 (1.03)	7.27 (1.76)	7.25 (1.85)	7.17 (1.60)	7.05 (1.81)	6.50 (2.39)	7.31 (1.70)
Pain catastrophizing (PCS) (0–52)^4^	23.66 (12.57)	23.87 (12.69)	26.33 (13.47)	22.84 (11.61)	23.53 (12.59)	22.76 (12.56)	23.41 (11.97)	18.75 (12.37)	23.84 (13.43)
Helplessness subscale (PCS) (0–24)^5^	11.08 (6.27)	11.26 (6.38)	12.67 (6.41)	10.70 (5.67)	10.89 (6.22)	10.61 (6.47)	10.80 (5.93)	8.25 (6.54)	11.18 (6.60)
Rumination subscale (PCS) (0–16)^6^	8.53 (4.32)	8.54 (4.35)	9.67 (4.84)	8.19 (4.09)	8.63 (4.30)	8.24 (4.25)	8.64 (4.19)	7.38 (4.03)	8.44 (4.56)
Magnification subscale (PCS) (0–12)^7^	4.18 (3.03)	4.19 (3.03)	4.00 (2.61)	4.03 (2.84)	4.18 (3.11)	3.91 (2.60)	4.15 (2.94)	3.13 (2.41)	4.30 (3.27)
General self‐efficacy (GSE) (0–40)^8^	27.94 (6.32)	27.81 (6.50)	27.83 (4.54)	28.15 (6.49)	28.22 (6.16)	28.09 (4.71)	28.01 (5.60)	27.00 (6.35)	28.09 (6.17)
Disability (ODI), baseline (0–100)^9^	42.82 (16.89)	43.08 (17.55)	52.88 (20.78)	41.27 (15.68)	43.82 (18.38)	40.98 (13.47)	41.27 (17.77)	35.11 (13.11)	43.81 (16.89)
Disability (ODI), 12 months (0–100)^10^	37.75 (17.40)	38.81 (17.68)	49.78 (7.65)	35.16 (15.02)	36.17 (16.22)	36.88 (13.23)	36.56 (18.49)	42.83 (18.31)	37.07 (17.21)

*Note*: Detailed test statistics for ANOVA comparisons between ICD‐11 categories: ^1^(F[7, 2312] = 15.08, *p* < 0.001), ^2^(F[7, 2424] = 0.85, *p* = 0.55), ^3^(F[7, 2542] = 1.17, *p* = 0.32), ^4^(F[7, 2471] = 0.42, *p* = 0.89), ^5^(F[7, 2471] = 0.73, *p* = 0.65), ^6^(F[7, 2442] = 0.38, *p* = 0.92), ^7^(F[7, 2454] = 0.30, *p* = 0.95), ^8^(F[7, 2529] = 0,25, *p* = 0.97), ^9^(F[7, 2791] = 1.72, *p* = 0.10), ^10^(F[7, 889] = 1,07, *p* = 0.38).

### 3.2 Differences across ICD‐11 chronic pain classifications

#### 3.2.1 Age and pain duration

The mean values of pain duration did not differ significantly across the eight ICD‐11 chronic pain classifications. There were significant differences in age at baseline. The mean age for the groups was from the highest to the lowest: 56 years for chronic secondary musculoskeletal pain, 53.8 for chronic cancer‐related pain, 53.4 for chronic neuropathic pain, 49.6 for unspecified chronic pain, 48.3 for chronic secondary visceral pain, 47.6 for chronic postsurgical or posttraumatic pain, 47.1 for chronic primary pain and 44.7 for chronic secondary headache or orofacial pain. As such, the biggest difference was between chronic secondary musculoskeletal pain and chronic secondary headache or orofacial pain where the first group was 11.3 years older than the latter.

#### 3.2.2 Coping expectancies, disability and pain intensity across ICD‐11 categories

Neither general self‐efficacy, pain catastrophizing nor its subscales helplessness, rumination and magnification showed any significant differences across the ICD‐11 chronic pain categories. The same was true for pain intensity as well as pain‐related disability at baseline and at 12‐month follow‐up where there were no significant differences in mean levels across the ICD‐11 chronic pain categories. The detailed parameters of the ANOVA test statistics are reported as footnotes in Table 1.

### 3.3 Associations of coping expectancies and disability at baseline

In the simple linear regression analyses, all included predictors had significant relationships to disability at baseline. The helplessness subscore showed the strongest association to disability compared to the other investigated variables, including the overall measure of pain catastrophizing and the additional subscales. Overall, higher levels of pain catastrophizing, helplessness, rumination and magnification predicted higher levels of disability.

In the simple linear regression analyses, helplessness explained 18% of the variance in disability at baseline. This number was 16% for the overall measure of pain catastrophizing, 9.7% for rumination and 12.5% for magnification.

Higher general self‐efficacy was significantly associated with lower disability at baseline, explaining 9.6% of the variance in the outcome. The detailed statistical test parameters of the simple regression analyses are reported as footnotes in Table 2.

**TABLE 2:** Multiple regression model of associations to disability at baseline in chronic pain patients

	*b*	SE	*β*	*p*‐value	95% CIs		*η*_p_^2^
Step 1							
Constant	39.69	1.93		**<0.001**	35.91	43.47	
Age at baseline	0.02	0.02	0.02	0.483	−0.03	0.07	0.000
Gender	0.49	0.85	0.01	0.563	−1.18	2.17	0.000
Step 2							
Constant	42.32	0.75		**<0.001**	36.72	47.92	
Age at baseline	0.02	0.02	0.02	0.303	−0.02	0.07	0.000
Gender	0.23	0.75	0.01	0.756	−1.24	1.71	0.000
Helplessness	1.04	0.07	0.38	**<0.001**	0.92	1.17	0.135
Self‐efficacy	−0.50	0.07	−0.22	**<0.001**	−0.63	−0.37	0.033
Step 3							
Constant	26.11	2.94		**<0.001**	20.34	31.88	
Age at baseline	0.01	0.02	0.01	0.443	−0.03	0.05	0.000
Gender	−0.29	0.71	−0.01	0.685	−1.70	1.11	0.000
Helplessness	0.72	0.07	0.26	**<0.001**	0.58	0.84	0.066
Self‐efficacy	−0.52	0.06	−0.19	**<0.001**	−0.64	−0.40	0.041
Pain intensity	2.91	0.22	0.30	**<0.001**	2.49	3.34	0.099
Pain duration	0.16	0.05	0.07	**0.001**	0.07	0.26	0.007
Step 4							
Constant	26.21	2.94		**<0.001**	20.44	31.98	
Age at baseline	0.02	0.02	0.02	0.475	−0.03	0.06	0.000
Gender	−0.31	0.71	−0.01	0.668	−1.71	1.09	0.000
Helplessness	0.71	0.07	0.26	**<0.001**	0.58	0.84	0.066
Self‐efficacy	−0.52	0.06	−0.20	**<0.001**	−0.64	−0.40	0.040
Pain intensity	2.92	0.22	0.30	**<0.001**	2.50	3.34	0.099
Pain duration	0.16	0.05	0.07	**0.001**	0.06	0.26	0.006
Primary versus secondary chronic pain	−1.01	0.73	0.03	0.165	−2.43	0.42	0.001

*Note*: *R* ^2^ for step 1: 0.00, *p* = 660, Δ*R* ^2^ for step 2: 0.23, *p* < 0.001, Δ*R* ^2^ for step 3: 0.08, *p* < 0.001, Δ*R* ^2^ for step 4: 0.00, *p* = 0.165.

*η*_p_^2^: partial eta squared. Test parameters for final multiple regression model (step 4): (*F*[71657] = 106.81, *p* < 0.001).

Test parameters for the simple linear regression analyses predicting disability at baseline: helplessness (*b* = 1.16, *t*[1, 2545] = 23.62, *p* < 0.001), pain catastrophizing (*b* = 0.54, t[1, 2545] = 22.03, *p* < 0.001), rumination (*b* = 1.23, *t*[1, 2516] = 16.41, *p* < 0.001), magnification (*b* = 2.00, *t*[1, 2528] = 19.02, *p* < 0.001), general self‐efficacy (*b* = −0.84, *t*[1, 2606] = −16.66, *p* < 0.001). Statistically significant results at *p* < .05 level are written in bold.

A hierarchical multiple regression model was built in order to investigate shared and unique contributions to the variance in disability at baseline (Table 2). First, the demographic variables age and gender were added to the model (step 1). These variables were not significantly associated with disability. In the next step, coping expectancies were included in the model (step 2). Since helplessness explained the greatest amount of variance in disability at baseline compared with the overall measure of pain catastrophizing and the additional subscales, this variable was selected for the multiple regression model. General self‐efficacy also showed a significant association with disability in the simple regression analysis and was included in this step along with helplessness. Furthermore, we controlled for the possible confounding variables pain intensity and pain duration (step 3). Both variables contributed significantly to the model, however, the effect of pain duration was minimal. Finally, a dummy variable of primary versus secondary chronic pain syndromes was added to the model (step 4). This did not result in any significant change. The final model (step 4) explained 31% of the variance in disability at baseline with a sufficient fit of the data. Here, helplessness, self‐efficacy, pain intensity and pain duration contributed significantly to disability at baseline at *p* ≤ 0.001.

### 3.4 Predictors of pain‐related disability at 12‐month follow‐up

Higher levels of helplessness were the strongest predictor of higher levels of disability at 12‐month follow‐up. Also, higher levels of pain catastrophizing, rumination and magnification significantly predicted higher levels of disability at 12 months. Finally, higher general self‐efficacy predicted lower levels of disability. In the simple regression analyses, helplessness explained 9.7% of variance in disability, pain catastrophizing 8%, magnification 6.3%, rumination 4.1% and self‐efficacy 8.3%. The detailed statistical test parameters of the simple regression analyses are reported as footnotes in Table 3.

**TABLE 3:** Multiple regression model of associations to disability at 12‐month follow‐up in chronic pain patients

	*b*	SE	*β*	*p*‐value	95% CIs		*η*_p_^2^
Step 1							
Constant	32.29	3.34		**<0.001**	25.73	38.84	
Age at baseline	0.07	0.05	0.03	0.122	−0.02	0.16	0.004
Gender	1.00	1.47	0.03	0.494	−1.88	3.89	0.001
Step 2							
Constant	34.55	5.85		**<0.001**	23.06	46.04	
Age at baseline	0.09	0.04	0.08	**0.040**	0.00	0.17	0.007
Gender	1.07	1.38	0.03	0.438	−1.64	3.78	0.001
Helplessness	0.81	0.12	0.28	**<0.001**	0.57	1.05	0.071
Self‐efficacy	−0.42	0.14	−0.13	**0.003**	−0.69	−0.14	0.015
Step 3							
Constant	7.19	4.57		0.116	−1.78	16.16	
Age at baseline	0.03	0.03	0.02	0.413	−0.04	0.09	0.001
Gender	0.72	1.01	0.02	0.473	−1.25	2.69	0.001
Helplessness	0.08	0.10	0.03	0.433	−0.11	0.27	0.001
Self‐efficacy	−0.19	0.10	−0.06	0.060	−0.40	0.01	0.006
Disability at baseline	0.76	0.04	0.70	**<0.001**	0.69	0.83	0.445
Pain intensity	0.08	0.32	0.01	0.798	−0.54	0.70	0.000
Pain duration	0.14	0.06	0.06	**0.031**	0.01	0.26	0.008
Step 4							
Constant	7.31	4.57		0.110	−1.66	16.28	
Age at baseline	0.03	0.03	0.03	0.307	−0.03	0.10	0.002
Gender	0.71	1.01	0.02	0.477	−1.26	2.69	0.001
Helplessness	0.07	0.10	0.03	0.449	−0.12	0.26	0.001
Self‐efficacy	−0.19	0.10	−0.06	0.062	−0.39	0.01	0.006
Disability at baseline	0.76	0.04	0.70	**<0.001**	0.69	0.83	0.445
Pain intensity	0.08	0.32	0.01	0.800	−0.54	0.70	0.000
Pain duration	0.13	0.06	0.06	**0.033**	0.01	0.26	0.008
Primary versus secondary chronic pain	−1.02	1.01	−0.03	0.316	−3.00	0.97	0.002

*Note*: *R* ^2^ for step 1: 0.01, *p* = 235, Δ*R* ^2^ for step 2: 0.12, *p* < 0.001, Δ*R* ^2^ for step 3: 0.41, *p* < 0.001, Δ*R* ^2^ for step 4: 0.00, *p* = 0.316,

η_p_^2^: partial eta squared. Test parameters for final multiple regression model (step 4): (*F*[8, 576] = 85.54, *p* < 0.001).

Test parameters for the simple linear regression analyses predicting disability at baseline: helplessness (*b* = 0.88, *t*[1, 869] = 9,68, *p* < 0.001), pain catastrophizing (*b* = 0.40, *t*[1, 869] = 8.70, *p* < 0.001), rumination (*b* = 0.80, *t*[1, 865] = 6.04, *p* < 0.001), magnification (*b* = 1.47, *t*[1, 867] = 7.61, *p* < 0.001), general self‐efficacy (*b* = −0.91, *t*[1, 880] = −8.90, *p* < 0.001). Statistically significant results at *p* < .05 level are written in bold.

The first step of the hierarchical model (Table 3) included the demographic variables age and gender (step 1) which did not predict disability at 12‐month follow‐up. Following, coping expectancies, that is helplessness and general self‐efficacy, were added (step 2), which both were small but significant predictors of the outcome. Adding the confounder variables, disability measured at baseline, pain intensity and pain duration significantly adjusted the model (step 3). As such, disability at baseline and, to a very small extent, pain duration, were the only significant predictors of disability at 12‐month follow‐up. Finally, including the primary versus secondary chronic pain distinction did not change the model and thus did not contribute to variance in disability at 12‐month follow‐up (step 4). The final model showed an acceptable fit and overall explained 54.3% of the variance in disability at 12‐month follow‐up.

### 3.5 Differences between responders and non‐responders of the follow‐up registration

Analyses were made to investigate systematic differences between responders and non‐responders on the 12‐month follow‐up registration that could increase the risk of bias. There were no demographic differences in terms of age or gender between participants who responded on the 12‐month follow‐up registration (*N* = 920) compared with those who did not respond (*N* = 1955). When the groups were compared in terms of baseline disability, pain intensity, self‐efficacy, pain catastrophizing and its subscales, some minor but statistically significant differences emerged. Responders on the follow‐up registration had a lower disability at baseline compared with the participants who did not respond. Responders had higher self‐efficacy than non‐responders and lower magnification. Effects were small, showing Cohen's *d*‐values close to zero (0.15, −0.16 and 0.12 respectively).

## 4 DISCUSSION

The aim of this paper was twofold; first, to investigate how expectancies of coping in the form of pain catastrophizing and self‐efficacy were distributed across the new ICD‐11 pain categories, and second, to assess how pain catastrophizing and self‐efficacy were associated with pain‐related disability cross‐sectional and longitudinally in a large and naturalistic pain clinic population. Rejecting our first hypothesis, the results showed no significant differences in coping expectancies across the diagnostic categories. No differences in terms of pain‐related disability or pain intensity across the categories were detected either. In terms of the second aim, our hypothesis was supported. At baseline, coping expectancies were associated with pain‐related disability. In fact, one of the subscales of pain catastrophizing—helplessness—showed the strongest positive relationship to disability compared with the overall pain catastrophizing construct and the other subscales. Longitudinally, coping expectancies did not remain significant as predictors of disability when controlling for baseline values of disability.

Contrary to our hypothesis, we found no difference between the eight ICD‐11 chronic pain categories on coping expectancies. Although meta‐analyses have concluded that levels of self‐efficacy and pain catastrophizing in chronic pain patients are not condition specific (Jackson et al., 2014; Martinez‐Calderon et al., 2019), a previous study had indicated that differences in self‐efficacy, helplessness and disability might exist between patients with fibromyalgia (chronic primary pain) and arthritis (chronic secondary pain) (Moyano et al., 2019). In light of the new ICD‐11 chronic pain classification system being implemented, it was critical to investigate whether coping expectancies were more strongly associated with chronic primary pain conditions compared with chronic secondary pain conditions. The role of coping expectancies is viewed as condition specific among many clinicians working with chronic pain patients, possibly due to the fact that the aetiology of primary chronic pain conditions such as fibromyalgia or irritable bowel syndrome (IBS) are still somewhat unknown. Conversely, the origin of the pain involved in chronic secondary pain conditions such as chronic post‐surgical pain (CPSP) or arthritis is more easily ascribed to physiological processes such as damage from surgery or inflammation, and could rely less on psychosocial factors. The results from this study make an important point in showing that levels of pain catastrophizing and self‐efficacy are similar across all the chronic pain diagnostic categories, and also showing that the distinction between primary and secondary chronic pain syndromes does not have any effect on pain‐related disability, neither cross‐sectional nor longitudinally. The results imply that chronic pain patients, independent of diagnosis, should be assessed for psychosocial contributors or consequences in relation to their disease. Importantly, the new ICD‐11 classification system for chronic pain permits this assessment through its extension codes for psychosocial factors. Defining a psychosocial extension code does not imply any causal links or aetiological assumptions, but should be used on the theoretical basis of chronic pain as a multifactorial, biopsychosocial phenomenon (Treede et al., 2019).

In the literature, robust, positive relationships between pain catastrophizing and disability have been established in a meta‐analysis, but more so for cross‐sectional than for longitudinal studies of patients with various musculoskeletal chronic pain conditions (Martinez‐Calderon et al., 2019). In our study, pain catastrophizing levels at baseline explained 8% of the variance in disability at 12‐month follow‐up in the simple regression analyses. However, when controlling for disability at baseline, pain catastrophizing was no longer a significant predictor of pain‐related disability at 12‐month follow‐up. Establishing these results in our large and diverse sample of chronic pain patients is an important contribution to the field in light of the scarcity of longitudinal studies (Martinez‐Calderon et al., 2019).

Finally, our study provided an opportunity to investigate general self‐efficacy as a coping expectancy impacting disability in chronic pain patients. General self‐efficacy is a broader, more trait‐like construct compared with pain‐specific self‐efficacy, which is more frequently investigated in chronic pain populations (Luszczynska, Gutiérrez‐Doña, et al., 2005). The effects we found are far smaller in size compared with those reported in a meta‐analysis on pain‐specific self‐efficacy and disability in chronic pain patients (Jackson et al., 2014). Hence, this could indicate that self‐efficacy beliefs are stronger predictors of pain outcomes when they are assessed in a more domain‐specific manner. On the other hand, Taylor et al. (2006) reported that general self‐efficacy had stronger associations to disability than health‐related self‐efficacy, in a general population sample with adults experiencing chronic pain—but only when pain had persisted for more than 1 year. In our study, the vast majority of patients had suffered from chronic pain for more than a year.

**FIGURE 1:**
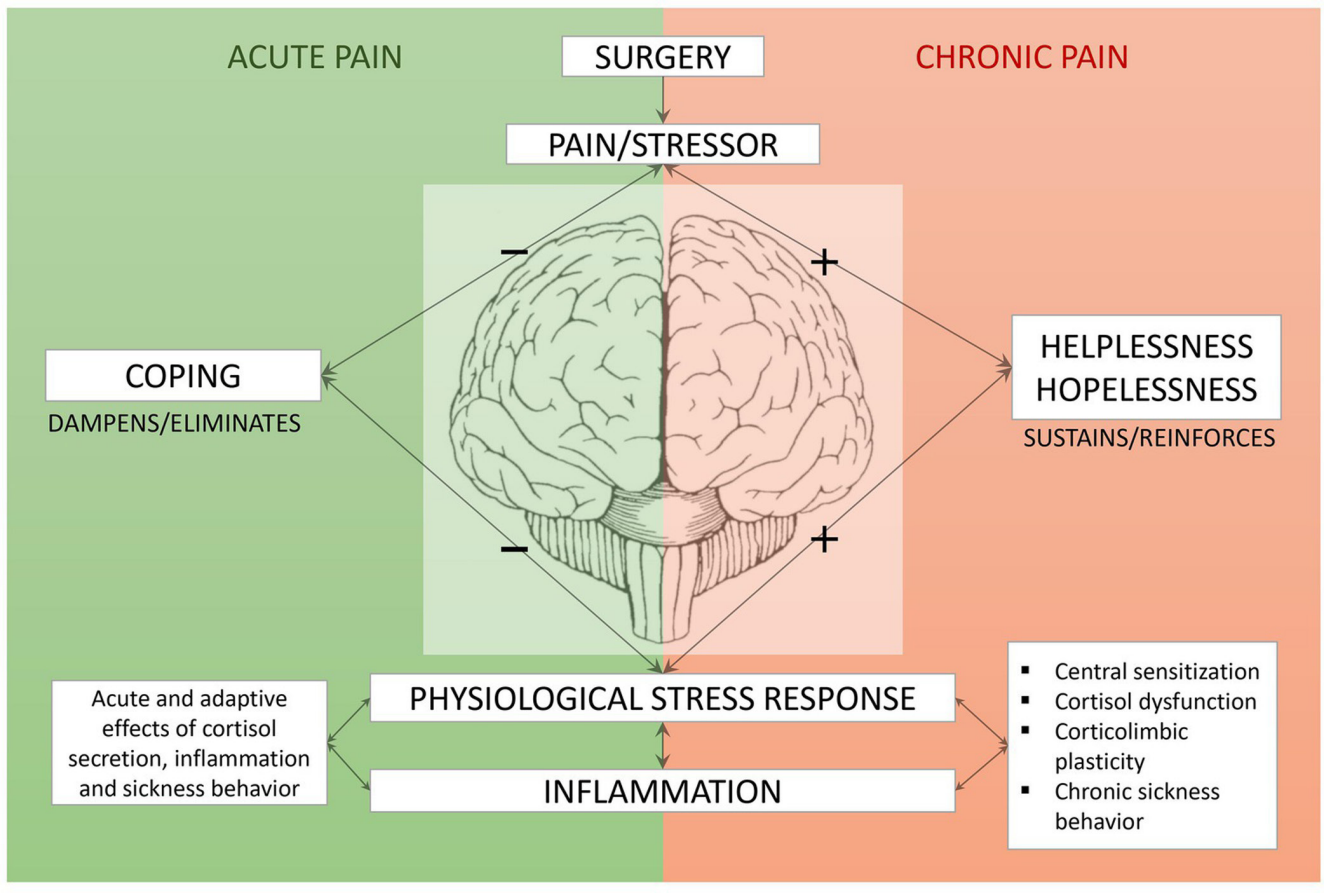
The SURGE‐model of chronic post‐surgical pain (©2021 Munk, Reme and Jacobsen, *front. Psychol*. 12:630422)

The cognitive activation theory of stress (CATS) is a psychobiological theory that links stress with illness and disease (Ursin & Eriksen, 2004). The theory states that expectancies of how one will be able to handle a stressful situation, predict the duration of the physiological stress response and ultimately the risk of developing negative health outcomes, that is chronic pain. Expectancies characterized by helplessness or hopelessness sustain the physiological stress response, while coping expectancies of being able to handle the situation with success dampen or eliminate it. The operationalization of coping in CATS is identical to generalized self‐efficacy beliefs (Ursin & Eriksen, 2004). Based on CATS, our research group recently developed the SURGE model of CPSP (Figure 1) (Munk et al., 2021). The SURGE model suggests that expectancies of helplessness, hopelessness or coping (i.e. general self‐efficacy) influence the risk of developing CPSP through neurobiological mechanisms such as central sensitization, neurotoxic effects of sustained secretion of cortisol, inflammation and sickness behaviour.

The theoretical perspectives of CATS and SURGE are interesting in light of the present findings since they link helplessness and general self‐efficacy to negative pain outcomes, and propose underlying neurobiological mechanisms. Our research group is currently testing the assumptions of SURGE in a randomized controlled trial aiming to prevent CPSP in women with breast cancer (ClinicalTrials.gov/NCT04518085). In addition, the creators of the OPR are planning to add a biobank to the registry (Granan et al., 2019) making it possible to test assumptions of the possible role(s) of stress hormones in chronic pain.

### 4.1 Strengths and limitations

A strength of the current study is the large sample size retrieved from a naturalistic setting of Norway's largest multidisciplinary pain clinic. The study includes validated questionnaires and longitudinal data and is theory driven. In future research, it could be relevant to include pain‐related distress as an outcome variable to capture the emotional aspects of pain. In the current study, this was outside the scope of the investigation. Results from the Pain Catastrophizing Scale have shown some cross‐cultural variations that should be taken into consideration. First, it is indicated that the clinical significance of pain catastrophizing in chronic pain patients varies between countries (Ikemoto et al., 2020). As an example, baseline mean scores of PCS in a chronic pain sample from Germany (Shaygan et al., 2018) were as low as 11.9 while the mean scores as high as 33.7 and 33.5 are seen in China (Man et al., 2007) and Japan (Inoue et al., 2017) respectively. Even more important for this study, factor analyses have shown cross‐cultural variations in the factor structure of the three subdomains (Fernandes et al., 2012; Van Damme et al., 2002). This decreases the generalizability and should be taken into consideration when comparing subscores across countries. A notable limitation is the response rate on 12‐month follow‐up, which suggest that the results from the longitudinal analyses should be interpreted with caution. However, the sensitivity analyses revealed that there were no meaningful differences in demographic or psychosocial variables between responders and non‐responders of the follow‐up registration. In our sample, some ICD‐11 diagnostic categories were considerably small in size. Thus, undetected differences in some of the smallest groups may exist. Overall, the minimal differences in demographic and clinical variables between responders and non‐responders and the large sample size strengthen the generalizability of the findings. Finally, the observational study design precludes any conclusions about causality.

## 5 CONCLUSION

Coping expectancies, hereunder pain catastrophizing and general self‐efficacy, are similar across the new ICD‐11 chronic pain categories. Likewise, levels of pain‐related disability, pain intensity and demographic characteristics do not differ across the categories either, except for age. Helplessness showed stronger associations to pain‐related disability than the overall measure of pain catastrophizing and its additional subscales, both cross‐sectional and longitudinally. Helplessness and general self‐efficacy are cross‐sectionally associated with pain‐related disability. Longitudinally, coping expectancies did not remain significant as predictors of disability when controlling for baseline values of disability. Patients with chronic pain, independent of diagnosis, may benefit from assessment of these psychosocial factors, and targeted interventions, such as CBT, should be considered.

## AUTHOR CONTRIBUTIONS

AM performed the literature review and wrote the first draft of the manuscript. AM, HBJ and SER all contributed to the conceptualization and writing of the final manuscript.

## CONFLICT OF INTEREST

The authors have no conflicts of interest to declare.

## Supporting information


Appendix S1
