# Supplementary material for: Coping expectancies and disability across the new ICD‐11 chronic pain categories: A large‐scale registry study
Source: Eur J Pain. 2022 May 31;26(7):1510–22. doi: 10.1002/ejp.1979 (PMC9543422; doi:10.1002/ejp.1979)
Supplement: Supplementary file 1 — Appendix S1 [file EJP-26-1510-s001.pdf]

Table: ICD-10 diagnosis of Oslo Pain Registry, converted to ICD-11 diagnosis*	
MG30.0 - Chronic primary pain	
F45.4	Persistent somatoform pain disorder
F48.0	Neurastenia
G44.0	Cluster headaches and other trigeminal autonomic cephalgias (TAC)
G44.2	Tension-type headache
G50.1	Atypical facial pain
G90.5	Complex regional pain syndrome I (CRPS I)
G90.6	Complex regional pain syndrome type II
G90.7	Complex regional pain syndrome, other and unspecified type
J32.0	Chronic maxillary sinusitis
J32.9	Chronic sinusitis, unspecified
K07.6	Disorder of the jaw
K07.9	Dentofacial anomaly, unspecified
K14.6	Glossodynia
K58.0	Irritable bowel syndrome with diarrhea
K58.3	Irritable bowel syndrome with mixed bowel habits [IBS-M]
K58.8	Other and unspecified irritable bowel syndrome
K58.9	Irritable bowel syndrome without diarrhea
K59.2	Neurogenic bowel, not elsewhere classified
M25.5	Pain in joint
M53.1	Cervicobrachial syndrome
M53.3	Sacrococcygeal disorders, not elsewhere classified
M54.2	Cervicalgia
M54.4	Lumbago with sciatica
M54.5	Low back pain
M54.6	Pain in thoracic spine
M54.9	Dorsalgia, unspecified
M75.1	Rotator cuff syndrome
M75.4	Impingement syndrome of shoulder
M75.9	Shoulder lesion, unspecified
M79.0	Rheumatism, unspecified
M79.1	Myalgia
M79.2	Neuralgia and neuritis, unspecified
M79.6	Pain in limb, hand, foot, fingers and toes
M79.7	Fibromyalgia
M79.9	Soft tissue disorder, unspecified
R07.0	Pain in throat
R07.2	Precordial pain
R07.3	Other chest pain
R07.4	Chest pain, unspecified
R10.1	Pain localized to upper abdomen
R10.2	Pelvic and perineal pain
R10.3	Pain localized to other parts of lower abdomen
R10.4	Other abdominal pain, not specified
R11	Nausea and vomiting
R20.1	Hypoesthesia of skin
R20.2	Paresthesia of skin
R20.8	Other disturbances of skin sensation

R25.8	Other abnormal involuntary movements
R29.8	Other symptoms and signs involving the nervous and musculoskeletal systems
R51	Headache
R52.0	Pain, unspecified
R52.9	Pain, unspecified
R53	Malaise and fatigue
R57.9	Shock, unspecified
R86.1	Abnormal level of hormones in specimens from male genital organs
MG30.1 - Chronic cancer-related pain	
C30	Malignant neoplasm of nasal cavity and middle ear
C34.9	Malignant neoplasm of unspecified part of bronchus or lung
C41.4	Malignant neoplasm of pelvic bones, sacrum and coccyx
C47.8	Malignant neoplasm of overlapping sites of peripheral nerves and autonomic nervous system
C49.4	Malignant neoplasm: Connective and soft tissue of abdomen
C50.9	Malignant neoplasm of breast of unspecified site
C53.9	Malignant neoplasm of cervix uteri, unspecified
C79.5	Secondary malignant neoplasm of bone and bone marrow
C83.3	Diffuse large B-cell lymphoma
C90.0	Multiple myeloma
C96.2	Malignant mast cell neoplasm
D48.0	Neoplasm of uncertain behavior of bone and articular cartilage
MG30.2 - Chronic postsurgical or posttraumatic	
F07.2	Postconcussional syndrome
G54.6	Phantom limb syndrome with pain
K91.1	Postgastric surgery syndromes
M80.0	Age-related osteoporosis with current pathological fracture
M96.0	Pseudarthrosis after fusion or arthrodesis
M96.1	Postlaminectomy syndrome, not elsewhere classified
S12.1	Fracture of second cervical vertebra
S13.4	Sprain of ligaments of cervical spine
S14.1	Other and unspecified injuries of cervical spinal cord
S14.3	Other and unspecified injuries of cervical spinal cord
S14.4	Injury of peripheral nerves of neck
S22.3	Fracture of rib
S24.1	Other and unspecified injuries of thoracic spinal cord
S24.3	Injury of peripheral nerves of thorax
S24.5	Injury of other nerves of thorax
S24.6	Injury of unspecified nerve of thorax
S34.1	Other and unspecified injury of lumbar and sacral spinal cord
S34.3	Injury of cauda equina
S34.6	Injury of peripheral nerve(s) of abdomen, lower back and pelvis
S42.0	Fracture of clavicle
S44.0	Injury of ulnar nerve at upper arm level
S44.5	Injury of cutaneous sensory nerve at shoulder and upper arm level
S54.1	Injury of median nerve at forearm level
S54.7	Injury of multiple nerves at forearm level
S58.9	Traumatic amputation of forearm, level unspecified
S64.1	Injury of median nerve at wrist and hand level
S64.3	Injury of digital nerve of thumb

S64.4	Injury of digital nerve of other and unspecified finger
S74.0	Injury of sciatic nerve at hip and thigh level
S74.1	Injury of femoral nerve at hip and thigh level
S74.2	Injury of cutaneous sensory nerve at hip and thigh level
S74.7	Multiple nerve lesions at hip or thigh level
S82.3	Fracture of lower end of tibia
S84.1	Injury of peroneal nerve at lower leg level
S84.2	Injury of cutaneous sensory nerve at lower leg level
S84.7	Multiple nerve lesions at knee or lower leg level
S84.8	Injury of other nerves at lower leg level
S94.1	Injury of medial plantar nerve
S94.3	Injury of cutaneous sensory nerve at ankle and foot level
S94.7	Injury of multiple nerves at ankle and foot level
T84.0	Mechanical complication of internal joint prosthesis
T84.2	Mechanical complication of internal fixation device of other bones
T86.9	Complication of unspecified transplanted organ and tissue
T87.3	Neuroma of amputation stump
T87.6	Other or unspecified complication in ambutaped lumb
T88.8	Other specified complications of surgical and medical care, not elsewhere classified
T88.9	Complication of surgical and medical care, unspecified
T90.5	Sequelae of intracranial lesion
T90.9	Sequelae of unspecified injury of head
T91.1	Sequelae of spinal racture
T91.2	Sequelae of other fracture in thorax or pelvis
T91.8	Sequelae of lesion in neck or body
T91.9	Sequelae of lesion in neck or body, unspecified
T92.3	Sequelae of dislocation, sprain and strain of upper limb
T92.4	Sequelae of dislocation, sprain and strain of upper limb
T92.9	Sequelae of lesion in over extremities, unspecified
T93.2	Sequelae of other fracture in lower extremities
T93.3	Sequelae of luxation or distortision in leg
T93.4	Sequelae of nerve lesion in lower extremities
T93.5	Sequelae of lesion in muscular/tendon in the lower extremities
T93.8	Sequelae of other lesion on under extremities
T93.9	Sequelae of lesion in lower extremities, unspecified
T94.0	Sequelae of injuries involving multiple body regions
T94.1	Sequelae of injuries involving multiple body regions
T95.8	Sequelae of other burn, corrosion or frost
T98.3	Sequelae of medical treatment, IKA
Z65.4	Victim of crime and terrorism
MG30.3 Chronic secondary musculoskeletal pain	
M06.0	Rheumatoid arthritis without rheumatoid factor
M08.1	Juvenile ankylosing spondylitis
M06.9	Rheumatoid arthritis, unspecified
M10.1	Lead-induced gout
M15.0	Primary generalized (osteo)arthritis
M15.8	Other polyarthrosis
M15.9	Polyosteoarthritis, unspecified

M16.0	Bilateral primary osteoarthritis of hip
M16.1	Unilateral primary osteoarthritis of hip
M16.9	Coxarthrosis, unspecified
M17.0	Bilateral primary osteoarthritis of knee
M17.1	Unilateral primary osteoarthritis of knee
M17.2	Bilateral post-traumatic osteoarthritis of knee
M17.3	Unilateral post-traumatic osteoarthritis of knee
M17.5	Other unilateral secondary osteoarthritis of knee
M17.9	Osteoarthritis of knee, unspecified
M18.0	Bilateral primary osteoarthritis of first carpometacarpal joints
M19.0	Primary arthrosis of other joints
M19.1	Post-traumatic arthrosis of other joints
M19.9	Arthrosis, unspecified
M23.9	Unspecified internal derangement of knee
M24.4	Recurrent dislocation of joint
M25.6	Stiffness of joint, not elsewhere classified
M32.9	Systemic lupus erythematosus, unspecified
M34.0	Progressive systemic sclerosis
M34.1	CR(E)ST syndrome
M34.8	Other forms of systemic sclerosis
M35.0	Sicca syndrome [Sjögren]
M35.1	Other overlap syndromes
M35.7	Hypermobility syndrome
M35.9	Systemic involvement of connective tissue, unspecified
M41.2	Other idiopathic scoliosis
M41.5	Other secondary scoliosis
M41.8	Other forms of scoliosis
M41.9	Scoliosis, unspecified
M45	Ankylosing spondylitis
M46.9	Unspecified inflammatory spondylopathy
M47.2	Other spondylosis with radiculopathy
M47.8	Other spondylosis
M48.0	Spinal stenosis
M48.5	Collapsed vertebra, not elsewhere classified
M48.8	Other specified spondylopathies
M50.0	Cervical disc disorder with myelopathy
M50.1	Cervical disc disorder with radiculopathy
M50.8	Other cervical disc disorders
M51.1	Thoracic, thoracolumbar and lumbosacral intervertebral disc disorders with radiculopathy
M51.3	Other thoracic, thoracolumbar and lumbosacral intervertebral disc degeneration
M54.0	Panniculitis affecting regions of neck and back
M54.3	Sciatica
M54.8	Other dorsalgia
M62.4	Contracture of muscle
M70.6	Trochanteric bursitis
M71.1	Other infective bursitis
M71.5	Other bursitis, not elsewhere classified
M72.2	Plantar fascial fibromatosis

M72.9	Fibroblastic disorder, unspecified
M75.0	Adhesive capsulitis of shoulder
M75.5	Bursitis of shoulder
M75.8	Other shoulder lesions
M76.8	Other specified enthesopathies of lower limb, excluding foot
M76.9	Unspecified enthesopathy, lower limb, excluding foot
M77.1	Lateral epicondylitis
M77.9	Enthesopathy, unspecified
M79.5	Residual foreign body in soft tissue
M80.1	Osteoporosis after ooforectomi with pathological fracture
M81.9	Osteoporosis, unspecified
M85.0	Fibrous dysplasia (monostotic)
M87.9	Osteonecrosis, unspecified
M89.0	Algoneurodystrophy
M89.4	Other hypertrophic osteoarthropathy
M89.6	Osteopathy after poliomyelitis
M91.1	Juvenile osteochondrosis of head of femur [Legg-Calvé-Perthes]
Q65.9	Congenital deformity of hip, unspecified
Q78.0	Osteogenesis imperfecta
Q78.6	Multiple congenital exostoses
Q79.6	Ehlers-Danlos syndrome
MG30.4 - Chronic secondary visceral pain	
I70.2	Atherosclerosis of arteries of extremities
I73.0	Raynaud's syndrome
I73.8	Other specified peripheral vascular diseases
K86.0	Alcohol-induced chronic pancreatitis
N18.3	Chronic kidney disease, stage 3
N30.1	Interstitial cystitis (chronic)
N41.1	Chronic prostatitis
N50.8	Other specified disorders of male genital organs
N80.9	Endometriosis, unspecified
N90.8	Other specified noninflammatory disorders of vulva and perineum
N99.0	Postprocedural (acute) (chronic) kidney failure
Q27.8	Other specified congenital malformations of peripheral vascular system
Q28.0	Arteriovenous malformation of precerebral vessels
Q28.9	Congenital malformation of peripheral vascular system, unspecified
Q87.4	Marfan syndrome
I20.9	Angina pectoris, unspecified
K50.0	Crohn's disease of small intestine
K50.8	Crohn's disease of both small and large intestine
K50.9	Crohn disease, unspecified
K51.0	Ulcerative (chronic) pancolitis
K51.9	Ulcerative colitis, unspecified
K86.1	Other chronic pancreatitis
N80.3	Endometriosis of pelvic peritoneum
MG30.5 - Chronic neuropathic pain	
B02	Zoster [herpes zoster]
B02.2	Zoster with other nervous system involvement

B02.8	Zoster with other complications
B91	Sequelae of poliomyelitis
D32.9	Benign neoplasm of meninges, unspecified
D33.0	Benign neoplasm of brain, supratentorial
D33.4	Benign neoplasm of spinal cord
E10.4	Type 1 diabetes mellitus with neurological complications
E10.8	Type 1 diabetes mellitus with unspecified complications
E10.9	Type 1 diabetes mellitus without complications
E11.4	Type 2 diabetes mellitus with neurological complications
G14	Postpolio syndrome
G20	Parkinson disease
G23.9	Degenerative disease of basal ganglia, unspecified
G24.8	Other dystonia
G25.8	Other specified extrapyramidal and movement disorders
G25.9	Extrapyramidal and movement disorder, unspecified
G43.1	Migraine with aura [classical migraine]
G43.3	Migraine with complications
G43.9	Migraine, unspecified
G44.1	Vascular headache, not elsewhere classified
G44.4	Drug-induced headache, not elsewhere classified
G50.8	Other disorders of trigeminal nerve
G50.9	Disorder of trigeminal nerve, unspecified
G51.8	Other disorders of facial nerve
G52.2	Disorders of vagus nerve
G54.0	Brachial plexus disorders
G54.4	Lumbosacral root disorders, not elsewhere classified
G54.7	Phantom limb syndrome without pain
G54.8	Other nerve root and plexus disorders
G54.9	Nerve root and plexus disorder, unspecified
G56.0	Carpal tunnel syndrome
G56.1	Other lesions of median nerve
G56.2	Lesion of ulnar nerve
G56.3	Lesion of radial nerve
G56.4	Causalgia of upper limb
G56.9	Unspecified mononeuropathy of upper limb
G57.0	Lesion of sciatic nerve
G57.1	Meralgia paresthetica
G57.3	Lesion of lateral popliteal nerve
G57.6	Lesion of plantar nerve
G57.8	Other specified mononeuropathies of lower limb
G57.9	Unspecified mononeuropathy of lower limb
G58.0	Intercostal neuropathy
G58.7	Mononeuritis multiplex
G58.8	Other specified mononeuropathies
G58.9	Mononeuropathy, unspecified
G60.0	Hereditary motor and sensory neuropathy
G61.9	Inflammatory polyneuropathy, unspecified
G62.0	Drug-induced polyneuropathy

G62.1	Alcoholic polyneuropathy
G62.2	Polyneuropathy due to other toxic agents
G71.0	Muscular dystrophy
G71.1	Myotonic disorders
G80.0	Spastic quadriplegic cerebral palsy
G80.9	Cerebral palsy, unspecified
G81.1	Spastic hemiplegia
G82.0	Flaccid paraplegia
G82.2	Paraplegia, unspecified
G82.4	Spastic tetraplegia
G82.5	Tetraplegia, unspecified
G83.4	Cauda equina syndrome
G95.0	Syringomyelia and syringobulbia
G95.1	Vascular myelopathies
G95.2	Other and unspecified cord compression
G95.9	Disease of spinal cord, unspecified
I61.9	Nontraumatic intracerebral hemorrhage, unspecified
I63.9	Cerebral infarction, unspecified
I67.9	Cerebrovascular disease, unspecified
I69.1	Sequelae of nontraumatic intracerebral hemorrhage
I69.3	Sequelae of cerebral infarction
I69.8	Sequelae of other cerebrovascular diseases
S04.3	Injury of trigeminal nerve
G35	Multiple sclerosis
G43.0	Migraine without aura
G50.0	Trigeminal neuralgia
G53.0	Paresis of multiple cranial nerves w. Infection
G62.8	Other specified polyneuropathies
G62.9	Polyneuropathy, unspecified
G63.2	Diabetic polyneuropathy
G95.8	Other specified diseases of spinal cord
G96.8	Other specified disorders of central nervous system
I69.4	Sequelae of apoplexia cerebri
M54.1	Radiculopathy
T11.3	Lesion on nerve at foot, unspecified
T13.3	Lesion on nerve on leg, unspecified
T91.3	Sequelae of lesion in spinal cord
MG30.6 Chronic secondary headache or orofacial pain	
G44.3	Post-traumatic headache
G44.8	Other specified headache syndromes
MG30.Y - Other specified chronic pain	
A60.0	Herpesviral infection of genitalia and urogenital tract
D57.1	Sickle-cell disease without crisis
D86.0	Sarcoidosis of lung
D86.8	Sarcoidosis of other sites
E76.2	Other mucopolysaccharidoses
E80.0	Hereditary erythropoietic porphyria
E85.0	Non-neuropathic heredofamilial amyloidosis

F10.1	Mental and behavioural disorders due to use of alcohol : harmful use
F19.1	Mental and behavioural disorders due to multiple drug use and use of other psychoactive substances
F331	Recurrent depressive disorder, current episode moderate
F43.1	Post-traumatic stress disorder
H15.9	Disorder of sclera, unspecified
H16.9	Keratitis, unspecified
H57.1	Ocular pain
H71	Cholesteatoma of middle ear
L40.5	Guttate psoriasis
L59.8	Other specified disorders of the skin and subcutaneous tissue related to radiation
L73.2	Hidradenitis suppurativa
L83	Acanthosis nigricans
L88	Pyoderma gangrenosum
L98.4	Chronic ulcer of skin, not elsewhere classified
Q85.0	Neurofibromatosis (nonmalignant)
Q98.8	Other specified sex chromosome abnormalities, male phenotype

MG30.Z - Chronic pain, unspecified	
R52.1	Malignant pain
R52.2	Other chronic pain
B03	Smallpox

*In version 1 of OPR clinicians did not electronically register ICD-11 main diagnosis. Based on registration of both ICD-10 and ICD-11 by clinicians of the pain department in OPR version 2, mapping between ICD-11 and ICD-10 was performed by registry personnel. On this basis ICD-10 codes have been converted to ICD-11. **Disclaimer:** This is not a complete conversion of all ICD-10 diagnosis to ICD-11. The conversion is based on Oslo University Hospital Pain Department's routines and culture for setting ICD-10 diagnosis.
